# Beyond genomic studies of congenital heart defects through systematic modelling and phenotyping

**DOI:** 10.1242/dmm.050913

**Published:** 2024-11-22

**Authors:** Deborah J. Henderson, Ahlam Alqahtani, Bill Chaudhry, Andrew Cook, Lorraine Eley, Lucile Houyel, Marina Hughes, Bernard Keavney, José Luis de la Pompa, John Sled, Nadine Spielmann, Lydia Teboul, Stephane Zaffran, Pleasantine Mill, Karen J. Liu

**Affiliations:** ^1^ MRC National Mouse Genetics Network, Congenital Anomalies Cluster, Harwell, OX11 0RD, UK; ^2^ Biosciences Institute, Newcastle University, Centre for Life, Newcastle upon Tyne NE1 3BZ, UK; ^3^ University College London, Zayed Centre for Research, London WC1N 1DZ, UK; ^4^ Congenital and Pediatric Cardiology Unit, M3C-Necker, Hôpital Universitaire Necker-Enfants Malades, APHP, Université Paris Cité, 149 Rue de Sèvres, 75015 Paris, France; ^5^ Cardiology Department, Norfolk and Norwich University Hospital, Norwich NR4 7UY, UK; ^6^ Division of Cardiovascular Sciences, School of Medical Sciences, Faculty of Biology, Medicine and Health, The University of Manchester, Manchester M13 9PT, UK; ^7^ NIHR Manchester Biomedical Research Centre, Manchester University NHS Foundation Trust, Manchester Academic Health Science Centre, Manchester M13 9PT, UK; ^8^ Intercellular Signaling in Cardiovascular Development and Disease Laboratory, Centro Nacional de Investigaciones Cardiovasculares (CNIC), Melchor Fernández Almagro 3, 28029 Madrid, Spain; ^9^ Ciber de Enfermedades Cardiovasculares, Instituto de Salud Carlos III, Melchor Fernández Almagro 3, 28029 Madrid, Spain; ^10^ Mouse Imaging Centre, Hospital for Sick Children, Toronto M5G 1XS, Canada. Department of Medical Biophysics, University of Toronto, Toronto M5G 1XS, Canada; ^11^ Institute of Experimental Genetics, German Mouse Clinic, Helmholtz Center Munich (GmbH), German Research Center for Environmental Health, D-85764 Neuherberg, Germany; ^12^ Mary Lyon Centre, MRC Harwell, Oxfordshire OX11 0RD, UK; ^13^ Aix Marseille Université, INSERM, Marseille Medical Genetics, U1251, 13005 Marseille, France; ^14^ MRC Human Genetics Unit, Institute for Genetics and Cancer, The University of Edinburgh, Edinburgh EH4 2XU, UK; ^15^ Centre for Craniofacial and Regenerative Biology, King's College London, London SE1 9RT, UK

**Keywords:** Congenital heart defect, Genetics, Gene variant, Human patient, Mouse model, Genomics, Disease modelling, Cardiac phenotyping, Developmental biology, Structural anomalies

## Abstract

Congenital heart defects (CHDs), the most common congenital anomalies, are considered to have a significant genetic component. However, despite considerable efforts to identify pathogenic genes in patients with CHDs, few gene variants have been proven as causal. The complexity of the genetic architecture underlying human CHDs likely contributes to this poor genetic discovery rate. However, several other factors are likely to contribute. For example, the level of patient phenotyping required for clinical care may be insufficient for research studies focused on mechanistic discovery. Although several hundred mouse gene knockouts have been described with CHDs, these are generally not phenotyped and described in the same way as CHDs in patients, and thus are not readily comparable. Moreover, most patients with CHDs carry variants of uncertain significance of crucial cardiac genes, further complicating comparisons between humans and mouse mutants. In spite of major advances in cardiac developmental biology over the past 25 years, these advances have not been well communicated to geneticists and cardiologists. As a consequence, the latest data from developmental biology are not always used in the design and interpretation of studies aimed at discovering the genetic causes of CHDs. In this Special Article, while considering other *in vitro* and *in vivo* models, we create a coherent framework for accurately modelling and phenotyping human CHDs in mice, thereby enhancing the translation of genetic and genomic studies into the causes of CHDs in patients.

## Teaser

**Summary:** Few genes have been proven as causal for congenital heart defects (CHDs). Here, we discuss ways to improve the molecular diagnosis of CHDs using mouse models to validate potential CHD genes.

## Introduction

As human disease genomics efforts continue to accelerate and improve, it seems increasingly likely that the causes of most congenital anomalies can be discovered. Congenital heart defects (CHDs; see Glossary, Box 1) are the most common congenital malformations, collectively affecting almost 1% of the population, and, by including bicuspid aortic valve (BAV; Box 1), this figure rises to ∼2.5% (Marelli et al., 2014; Tutar et al., 2005). CHDs range in severity from malformations such as hypoplastic left heart syndrome (HLHS; Box 1), which is incompatible with life without surgery, to less severe forms such as ventricular septal defects (VSDs; Box 1), some of which will be resolved spontaneously as the patient grows. The majority of CHDs affect the valves (Box 1) and septa (Box 1) of the heart, and, in doing so, disrupt the flow of blood around the body. Surgery is the most common treatment for moderate to severe CHDs, although in many cases this does not provide a cure and the heart will begin to decompensate over time, requiring further medical and/or surgical treatment, sometimes including transplantation. Thus, CHDs are frequently a lifelong burden for the patients, their families and the health system. Although many genetic and genomic studies have been carried out to identify genes that cause CHDs, relatively few targets have been found and even fewer have been proven to be causative. Complex reasons exist for this. At the level of the patient, the degree of phenotypic specification needed for clinical care may be insufficient for research studies focused on mechanistic discovery. The disconnect between cardiac developmental biologists and geneticists, and especially clinicians, has meant that advances in developmental biology that would be helpful for planning, conducting and interpreting studies with CHD patients are not well communicated. Added to this are the combined challenges of filtering genomic data to identify specific CHD-causing gene variants (Box 1), then validating and proving causation for these targets.

**Box 1. Glossary**

**Alternative splicing:** a process during gene expression by which certain exons are included or skipped in the mature mRNA transcript. This leads to the expression of distinct splice variant proteins from a single gene.

**Aneuploidy:** presence of an abnormal number of chromosomes. Down syndrome, where patients have three copies of chromosome 21, rather than the usual two copies, is an example of aneuploidy.

**Aortopathy:** degeneration of the arterial wall that can lead to aneurysm (abnormal ballooning) and dissection (tearing) of the aortic wall.

**Arterial valve:** valve that occurs in the aorta and pulmonary trunks as they leave the ventricles. These valves maintain unidirectional flow of blood from the heart into the circulation.

**Atria:** the collecting chambers of the heart where blood enters first from the lungs and the body.

**Atrial septal defect (ASD):** a common CHD that results from an abnormal opening between the two atria.

**Atrioventricular canal:** the region between the atria and the ventricles where the atrial and ventricular septa meet with the atrioventricular (mitral and tricuspid) valves.

**Atrioventricular septal defect (AVSD):** a serious form of CHD in which there is failure to properly separate the atria from the ventricles. AVSD is the most common CHD found in Down syndrome.

**Bicuspid aortic valve (BAV):** a common form of CHD in which there are two leaflets (moving components) of the aortic valve rather than the usual three. This is generally a mild malformation if found in isolation but can be associated with more severe forms of CHD. It is also associated with thoracic aortic aneurysm and predisposes patients to calcific aortic valve disease in later life.

**Cardiac cushions:** structures in the embryonic heart that form the cardiac valves and membranous septa.

**Cardiogenesis:** the process of heart formation in the embryo.

**Common arterial trunk:** a serious form of CHD in which the primitive outflow tract in the embryo does not divide to form the separate aorta and pulmonary trunk.

**Complex CHD:** occurrence of a group of CHDs within a single patient that may affect different structures within the heart.

**Congenital heart defect (CHD):** any abnormality in the structure of the heart that is apparent at birth. In many cases these affect the normal functioning of the heart.

**Copy number variation (CNV):** a situation in which the number of copies of a region of DNA varies between the genomes of different individuals. The length of the CNV can vary between a handful and thousands of copies depending on the region in question. CNVs are associated with both classical Mendelian and complex disease.

**CRISpr MEdiated REarrangement (CRISMERE):** a CRISPR/Cas9-based technique for generating large genomic variants (insertions, deletions, duplications) in a range of species.

**CRISPR-Cas9:** a powerful genome-editing technology for precise targeting of DNA sequences in a range of organisms.

***De novo*:** literally, ‘rising anew’. In the context of gene variants, means an alteration to the structure of DNA that occurs in the egg or sperm of the parents, or in the newly fertilised egg, and thus is first apparent in the offspring.

**Familial CHD:** a CHD that is inherited and can be seen in different generations of the same family.

**Gene burden testing:** a method for combining all information about variability within a gene into a single ‘burden score’. They are usually used to identify enrichment of rare harmful variants in specific genes.

**Gene Ontology (GO) analysis:** a bioinformatic process that can be used to determine the biological processes, molecular functions and cellular components of a gene. It is frequently used to gain evidence that a gene may be associated with a specific disease.

**Genetic rescue experiments:** an injected gene variant is used to rescue a null phenotype, whereby rescue suggests that the variant has normal function and non-rescue suggests that the variant disrupts gene function.

**Gene variant:** variations in the sequence of a gene that may disrupt gene function (pathogenic) in some cases.

**Genome-wide association study (GWAS):** a research tool that can be used to identify genomic variants in large populations that are statistically associated with a particular phenotype or disease.

**Human induced pluripotent stem cell (hiPSC):** a type of pluripotent cell (that is itself immature but can give rise to many other cell types) that is produced by reprogramming human differentiated cells.

**Hypoplastic left heart syndrome (HLHS):** a serious form of CHD characterised by a small (hypoplastic) left ventricle, abnormalities of the mitral and aortic valves, and a hypoplastic aorta. There appear to be at least three subtypes of HLHS, and, in the majority of cases, it is only compatible with life with immediate surgery.

**Indel:** a general term for insertion, deletion, or insertion and deletion of short regions of DNA.

**Isolated CHD:** a CHD that occurs with no other congenital abnormalities or disease associations.

**Mendelian inheritance:** inheritance patterns that follow the laws laid out by Gregor Mendel. This involves a wild-type (normal functional) allele and a mutated (disease) allele, which can have dominant (will cause disease if there is only one copy) or recessive (two copies required for the disease to manifest) action. This type of inheritance is rare for CHD.

**Mitral atresia:** a form of CHD in which the mitral valve, between the left atrium and left ventricle, has no opening so that no blood can flow through it.

**Morphogenetic:** the processes that lead to form and structure (shape) during embryonic development.

**Neural crest cells (NCCs):** multipotent cells arising in the early embryo that migrate to many locations in the body, including the heart and differentiate to a range of cell types.

**Next-generation sequencing (NGS):** a technique that allows researchers to sequence millions of DNA fragments simultaneously, allowing whole exomes (the coding part of the genome) and genomes (the entire genetic material) to be sequenced very rapidly.

**Nonsense-mediated decay (NMD):** a cellular surveillance mechanism that removes mRNA fragments that have premature stop codons within them. This maximises error-free gene expression.

**Outflow tract:** a structure in the embryonic heart that carries blood from the ventricles to the blood vessels that carry the blood around the body. It divides to form the roots of the aorta and pulmonary arteries.

**Non-coding sequence:** part of the genome that does not code for proteins.

**Partial penetrance:** penetrance is the likelihood that a phenotype will present itself in an individual. Partial penetrance occurs when some individuals that carry a particular pathogenic variant do not manifest the phenotype.

**Pathogenic variant:** a gene variant that predisposes the individual to malformation or disease.

**Persistent ductus arteriosus:** the abnormal persistence of a blood vessel that carries blood between the pulmonary trunk and the descending aorta before breathing begins at birth. In the normal situation, this blood vessel will close in the first few days following birth.

**Phenotype:** the observable traits within an individual. This compares to genotype, which is the set of genes encoded in DNA of an individual. The phenotype is determined by the genotype.

**Proband:** the first member of a family to be identified with a particular malformation or disease.

**RNA sequencing (RNAseq):** a transcriptomic technique for sequencing the entire RNA profile of a cell, tissue or organism.

**Second heart field (SHF):** multipotent cell population that originates in the splanchnic mesoderm and adds to the poles of the heart tube to form the outflow tract, right ventricle and much of the atria.

**Septa:** structures that separate the chambers and outflow tract of the heart.

**Single-cell RNA sequencing (scRNAseq):** a technique for sequencing single cells to reveal their mRNA profile (transcriptome).

**Spatial transcriptomics:** methodologies for assigning cell characteristics (identified by the mRNA that they express) to their position in tissues based on histological sections.

**Sporadic CHD:** a CHD that does not appear to be inherited within a family.

**Syndromic CHD:** a CHD that occurs together with other developmental abnormalities or disease manifestations affecting other parts of the body.

**Teleost genome duplication event:** a duplication of the genome of bony fishes that is thought to have occurred ∼320 million years ago.

**Teratogenic:** a teratogen is a factor, often a drug or chemical, that cause birth defects when the mother encounters it during pregnancy. Teratogenic means that the factor has the capacity to act as a teratogen.

**Thoracic aortic aneurysm:** weakness in the wall of the aorta within the chest that leads to a bulge in the arterial wall.

**Transcriptome:** full set of RNA expressed within an individual. Frequently used to refer to all the mRNA expressed within a cell type, tissue or organ.

**Valves:** structures that open and close as the heart beats to facilitate unidirectional flow of blood through the heart and into the body.

**Ventricles:** the main pumping chambers of the heart.

**Ventricular septal defect (VSD):** a type of CHD in which there is a communication (hole) between the two ventricles. The communication can be in a variety of positions and can affect the muscular or membranous part of the ventricular septum.

**Variant of uncertain significance (VUS):** a gene variant for which effect is uncertain or unknown.

Small-animal models – principally mice – have proven crucial for the identification of genes involved in cardiogenesis (Box 1), and a large number of genes essential for normal heart development are now known (Williams et al., 2019). In addition to generating total or conditional knockouts, it is now possible to generate mice carrying patient variants, with obvious utility for proving the link between the variant and the patient phenotype (Box 1), and also for investigating the timing of onset of disease and disease progression. Although mice and humans are highly similar in cardiac anatomy and function, there are many challenges in the use of mice to model human CHDs. These include, for instance, difficulties in engineering implicated genetic changes, the small size of the mouse heart, which complicates phenotyping, and the lack of standardised approaches for phenotyping and reporting the cardiac malformations.

To discuss these challenges within the community, the National Mouse Genetics Network Congenital Anomalies cluster hosted a workshop (funded by the Medical Research Council and the British Heart Foundation), ‘CHD: From gene variant to mouse model’, in November 2023, with over 70 attendees in person and 100 online. Based on presentations and discussions, we have created this Special Article as a framework for accurately modelling and phenotyping human CHDs in animal models, thereby enhancing the translation of genetic and genomic studies into the causes of CHDs in patients. Although this network focuses principally on modelling CHDs in mice, we also discuss some complementary approaches to modelling CHDs.

## Progress towards understanding the genetic causes of CHDs

Some cardiac malformations are attributable to teratogenic (Box 1) environmental issues, such as maternal diabetes or drugs (e.g. sodium valproate), or to other factors such as placental dysfunction. A detailed description of the environmental factors associated with CHDs is beyond the scope of this article, although the subject has been comprehensively reviewed in recent times (e.g. Kalisch-Smith et al., 2020; Boyd et al., 2022; Camm et al., 2018; Andescavage and Limperopoulos, 2021).

Although a genetic cause is proposed for the majority of CHDs, simple Mendelian inheritance (Box 1) of a gene through multiple generations of a family is rarely observed. Recurrence risks within families vary considerably for different types of CHD, reported as 24.3% for atrioventricular septal defect (AVSD; Box 1) but only 3.4% for VSD. Notably, the overall recurrence risk for CHD in a first-degree relative of a proband (Box 1) is only 3.45% (95% confidence interval 3.15-3.78) (Øyen et al., 2009, 2010). This suggests that although there are genetic influences, these are complex. Given this complexity, it is useful to categorise CHDs as familial (Box 1) or sporadic (Box 1), where familial cases have the greatest likelihood of achieving a genetic diagnosis. Familial cases showing a classical Mendelian inheritance, however, only represent ∼2% of cases and often involve milder survivable malformations, such as atrial septal defects (ASDs; Box 1) or persistent ductus arteriosus (Box 1). Even within a single family, partial penetrance (Box 1) of CHDs is common, often with a great degree of phenotypic variation between patients (Pierpont et al., 2018). Extended, multigenerational, family histories, with cardiac imaging, are rarely available. Moreover, until recently, babies born with severe CHD would die shortly after birth, frequently undiagnosed, and thus are excluded from further consideration. Therefore, even in familial cases, identifying the causative gene is not straightforward. In the more common, sporadic, category of CHD, gene disruption may have arisen *de novo* (Box 1) in the patient or one of their parents, or partial penetrance and/or phenotypic variation may make the inheritance pattern so unclear that it is not recognised as familial. In these cases, the chances of making a genetic diagnosis are much reduced.

Although most CHDs arise as isolated (Box 1) malformations, with the patient being otherwise healthy (Garg, 2006), many congenital syndromes (including Down syndrome, 22q11 deletion syndrome and some ciliopathies) include CHDs, making up ∼20% of CHD cases. Of these, ∼15% are related to aneuploidy (Box 1), with a further 20% related to copy number variations (CNVs; Box 1) of shorter regions of chromosomes (Soemedi et al., 2012a,b; reviewed in Pierpont et al., 2018). Long-range sequencing studies may show a yet higher contribution from CNVs to CHD incidence. Notably, there has been significant success in identifying single-gene causes of syndromic CHD (Box 1), which, at 30%, greatly exceeds that seen in sporadic CHD, for which a gene has been identified in only ∼5% of cases (reviewed in Wilde et al., 2022). Some caution should be applied to these proportions, as in this context ‘syndromic’ generally means the co-presentation of CHD with other developmental abnormalities, primarily neurodevelopmental impairment or extracardiac malformations (e.g. Homsy et al., 2015; Sifrim et al., 2016; Liu et al., 2020a,b), rather than the diagnosis of a recognised genetic syndrome (examples of such syndromes that typically involve CHD would include Noonan, Alagille and Kabuki syndromes, among many others). Nevertheless, the higher incidence of gene discovery when CHD co-exists with other developmental anomalies suggests that these cases are more genetically tractable, and are more likely to result from the action of genetic variants with large effects on CHD risk.

Despite the relative success with identifying genes involved in syndromic CHDs, as almost 80% of the CHD burden occurs as isolated sporadic defects, this means that the overall genetic explanation rate for CHD is low (at only 7%), which is much lower than most other common congenital anomalies. Indeed, the Genomics England panel for familial non-syndromic CHD has only 34 genes rated as ‘diagnostic level’, several of which are also implicated in syndromic CHDs. This highlights that, despite many genes being implicated in CHDs, very few have enough evidence to be useful for diagnostic screening. There are several possible explanations for why few gene variants are confidently designated as causal of CHDs. Studies in animal models have shown that disruption of different morphogenetic (Box 1) pathways can result in the ‘same’ CHD, and mutations affecting a single gene can result in a spectrum of malformation (Bajolle et al., 2009). This leads to the issue of ‘lumping’, where potentially diverse CHD phenotypes are placed together, or ‘splitting’, where CHDs are strictly matched for phenotype, within genetic studies (Thaxton et al., 2022). As an example, we do not know whether the same morphogenetic pathways are implicated in the different types of the complex CHD (Box 1) HLHS, and thus whether they should be ‘lumped’ together in genetic studies. In previous studies, highly heterogeneous CHD patient phenotypes have been combined, particularly for genome-wide approaches that require large numbers of patients for effective analysis. However, the more refinements made to the phenotype inclusion criteria, the fewer patients and lower statistical power available for each sub-phenotype. Although it is inevitable that the relative scarcity of some CHD phenotypes means that some degree of phenotypic aggregation is required to achieve statistical power, informed ‘lumping’, based on sound developmental biology data from animal models, might help to identify cohorts with related developmental aetiologies and improve the outcome of these types of studies. While this highlights issues surrounding the specificity of phenotypes, it could also be that pathogenic variants (Box 1) in more than one gene are necessary to lead to a given phenotype, known as oligogenic (if a few genes) or polygenic (if many genes) inheritance (e.g. Priest et al., 2016).

To add to the issues of combining CHD patients for genetic studies, once next-generation sequencing (NGS; Box 1) is carried out, important information may be disregarded. For example, alternative splicing (Box 1) is not universally taken into consideration and could account for some undiagnosed cases (Schoch et al., 2020). In addition, most of the genome (90%) contains non-coding sequences (Box 1) that may have a regulatory effect on nearby or distant genes, as revealed by a growing number of studies in mice (e.g. Bosada et al., 2023; Luna-Zurita et al., 2023), but these sequences are not currently captured in panel- or whole-genome-based approaches. This all highlights the complexity of defining the genetic architecture underlying CHD. This is not to say that there have been no successes – for example, the identification of *NKX2.5* (*NKX2-5*) and *GATA4* variants as causal of CHD – but the success rate is low in comparison with, for example, neurodevelopmental genes causing intellectual disability. Indeed, in the 100,000 Genomes Project, which takes a whole-genome approach, primary CHD families have one of the lowest solved rates for the entire programme. Notably, where a causative variant has been identified, these are rarely on the CHD panel. This means that the majority of potentially causative identified gene variants remain designated as ‘variants of uncertain significance’ (VUS; Box 1), highlighting the importance of validation studies.

There is no doubt that larger genetic studies incorporating more CHD patients are required. We estimate that ∼5000 CHD patients have had their genomes examined in the published literature, compared to hundreds of thousands for patients with coronary artery disease or hypertension. Thus, larger studies will undoubtedly reveal currently unidentified CHD-causing genes or loci. Genome-wide association studies (GWAS; Box 1) in CHD were first performed over 10 years ago and showed evidence for common genetic variants influencing CHD (Cordell et al., 2013), and more recent studies have confirmed that common variants play a significant part in CHD risk (Škorić-Milosavljević et al., 2022). Genomic regions identified by GWAS generally encompass multiple genes, and, in the absence of a particularly strong regional candidate, identification of a causal gene may be extremely challenging. Extrapolation from other conditions of similar heritability to CHD would suggest that the number of regions identified at statistical significance will scale commensurate to study size, although the effect size of individual loci will be smaller, with an increased number of identified regions. To generate potentially clinically useful polygenic risk scores, it would be anticipated that tens of thousands of patients would need to be studied. With regard to studies focused on rare variants, an increased availability of sequenced probands could facilitate more powerful analytical approaches based on gene burden testing (Box 1).

However, conducting larger studies alone will not resolve the issue. Deeper phenotyping and standardised coding of CHDs by cardiologists could lead to more focused and precise genetic studies that will avoid ‘lumping’ together groups of heterogeneous CHDs. More detailed compilation of family histories, with imaging data where possible and including the recording of neonatal death, could increase identification of familial cases. Biobanks undoubtedly have a role to play, with data from UK Biobank used recently to identify rare variants in *GATA6* associated with CHD (Williams et al., 2022). However, these authors drew attention to the limited family history and phenotyping typically available in large-scale biobank projects. Better awareness and understanding of advances in studying cardiac development in animal models, coupled with ongoing CHD clinical genetics, will lead to better design of future genomic studies and more information for patients, their families and their clinicians. Strengthening the connection between data obtained from humans and animal models, particularly mice, is imperative for comprehensive analysis of human CHDs.

## Phenotyping cardiovascular defects in patients as a basis for what we could/should do in mice

The identification of patient cohorts for genomic studies relies on two important factors: first, the accurate description of the patient's cardiac phenotype; and second, the recording of it in a format that can be readily understood and utilised by other clinicians, cardiac developmental biologists and geneticists. The acquisition of these accurate phenotyping data, including cardiac imaging, means that even as concepts and classifications change, the data continue to be accessible and useable for reinterpretation. An accurate description of the patient's cardiac phenotype is thus pivotal in order to group patients with the same characteristics.

Several issues hinder the characterisation of CHDs. With hundreds of different lesions described, in various combinations, and with varying severity, different nomenclatures have been developed to describe both the normal and abnormal heart. Thus, devising a system that is understood and accepted by all clinicians, and that is understandable to scientists, is necessary to deconvolve CHDs. It seems logical that if mice are to be used to model CHDs and to validate human CHD variants, then there needs to be a phenotyping and nomenclature system in place that, ideally, can be used interchangeably between human and mouse, or at the minimum, can be used to compare studies between mouse and human.

Although the complexity of CHDs makes accurate phenotyping challenging, methodologies have been established to systematically describe the normal and abnormal human heart. These approaches tend to rely on the division of the heart into segments [e.g. atria (Box 1), ventricles (Box 1), the great arteries], and this is followed by a description of the relationships between them. Different methodologies have become popular on different sides of the Atlantic, with the Van Praagh ‘Segmental Classification’ popular in the USA, and the ‘Sequential Segmental Analysis’ method championed by Anderson and colleagues in Europe (Van Praagh, 1972; Shinebourne et al., 1976). Although similar in approach, these guidelines use different nomenclatures and place an emphasis on different aspects of anatomy. For the purposes of this article, the details of the differences are not relevant, although the universal adoption of one method would significantly align efforts in the characterisation of CHD. Crucially, both methods, if followed, offer a relatively simple and systematic framework for describing all the cardiac malformations present within a patient's heart, allowing comparison with those observed in other patients.

Once a method for phenotyping the malformed heart is agreed upon, a classification system for naming the defects observed becomes essential. The first major international collaboration for disease classification was published in 1900, called the International Classification of Diseases (ICD), Version 1 (ICD-1), and was designed to promote international comparability in the collection, processing, classification and presentation of mortality statistics. ICD-1 included only a single item or code for congenital heart disease. By 2021, a common international code for congenital heart disease had been developed: the International Paediatric and Congenital Cardiac Code (IPCCC). The IPCCC gives each structural term a descriptive name, a definition and a unique six-digit numerical code. Work over many years has produced an extremely granular version, containing more than 10,000 terms, and a slightly truncated version, with ∼2000 terms. Crucially, in recent years, fundamental elements of the IPCCC have been incorporated into the latest revision of the ICD, further truncating the number of terms, aiming for greater standardisation, efficiency and simplicity, while still encompassing the spectrum of congenital cardiac malformations: this is IPCCC ICD-11 (Jacobs et al., 2021). The IPCCC ICD-11 thus describes the diagnostic hierarchy, built upon a sequential segmental method, and includes the definitions and codes for 367 terms. However, the formulation of these internationally accepted coding systems has not solved all the problems; for example, the IPCCC ICD-11 does not include other organs that may be implicated in syndromic CHD.

A further complication is that geneticists have evolved their own coding system – Human Phenotyping Ontology (HPO) – that uses a hierarchy of standardised terms to characterise human phenotypes. It thus acts as a resource for describing a patient's phenotype consistently and accurately, and as it utilises information from Orphanet, DECIPHER and Online Mendelian Inheritance in Man (OMIM), enables a link between specific malformations and/or patterns of disease and causal genes. As well as providing data on isolated CHDs, HPO also describes CHDs occurring within syndromes, and thus brings together malformations in disparate body systems. However, the number of terms used to describe CHDs are limited (there are nine for VSD compared to 32 in IPCCC ICD-11) and are not necessarily those that would be used by a cardiologist. Thus, HPO is not a substitute for the detailed coding described by IPCCC ICD-11. Similarly, useful resources such as CHDgene, which contains essential information about genes that have been reproducibly shown to cause CHD when mutated in humans (Yang et al., 2022), are also limited by the terms used to describe clinical phenotypes. Other resources such as those available at Alliance of Genome Resources and the Monarch Initiative are good for linking of genes to phenotypes but have only rudimentary classifications of CHDs. Linking IPCCC ICD-11 to HPO terms, and potentially other resources, would be very useful to the scientist dealing with genetic data from CHD patients and to the developmental biologist studying CHDs and/or modelling CHD variants.

Whatever the coding system employed, implementation in the clinical and scientific realm is often inconsistent, resulting in poor-quality data. There is intrinsic complexity and delay in incorporating the classification structure into the computation of administrative and patient information systems, and there is vague and incomplete recording of structured classification by clinical teams within patient records. These factors obfuscate accurate genetic correlation of cardiac structural malformations in humans, and hamper efforts to reliably correlate models of genetic disease with patients. Furthermore, detailed phenotyping is not cost-free, either resource-wise or scientifically.

Despite these issues, the situation in humans is more advanced compared to the situation for animal models. Currently there is no universally (or even well-recognised) approach to phenotyping mice for CHDs, despite there being many mouse mutants available with a wide range of phenotypes. As such, mouse mutants modelling CHDs are not generally described in a systematic, logical manner, as is seen with segmental analysis of human CHDs. Although there are many groups that show good practice, usually working alongside experienced human cardiac anatomists, this is not always the case, and many CHD models are inaccurately or incompletely described, with obvious implications for the interpretation of genetic studies. Overwhelmingly, the identification of CHD in mouse is simplistic, ignoring the type and position of malformation, which may involve crucial developmental and haemodynamic implications. For example, whereas IPCCC ICD-11 has 32 descriptors (codes) for malformations of the ventricular septum, in mouse there is usually only one (VSD), with additional descriptions, such as muscular or peri-membranous, that give additional information about type or position, being less common. The Mouse Genome Informatics (MGI) Mammalian Phenotype Browser, the most comprehensive and accessible database of phenotyped mouse mutants, takes a hierarchical approach to describing malformations – including CHDs – and utilises some terms that are used clinically. Indeed, the browser lists four terms for VSD and several hundred mouse mutants described as having these malformations. While acknowledging the huge value of this resource, the browser is limited by the descriptions of CHDs produced by the originating authors for each mouse mutant, and this generally makes a meaningful comparison with human malformations difficult.

Immediate actions are needed to prospectively develop systematic and logical coding and classification systems for mouse models of CHDs that can be consistently applied. Potentially, a version of segmental analysis that uses a critical subset of terms, similar to those used in human disease, could be devised to use in mice. This would allow clinicians, and human and mouse genetic scientists to efficiently understand each other's respective representations. Ideally, simple descriptive language describing the cardinal features of phenotypes should be maintained in a mouse coding system, rather than perpetuating historic, vague terms or acronyms that currently occur within the human system. Thus, the goal will be to find a ‘sweet spot’, where there are enough phenotyping data from patients and mouse mutants to make a meaningful comparison possible, but without being dauntingly complicated. In practice, it is likely that this will be an iterative process and will need to be extensively bench checked before roll-out. However, with these principles in mind, acknowledging and correcting some of the disadvantages of the current human classification systems, and with open collaboration and communication, there is great potential to achieve a standardised, inclusive and internationally accepted classification and coding system for CHD mouse models. This is a future goal of the authors of this article (Box 2).

**Box 2. Recommendations for congenital heart defect (CHD) research – next steps and milestones**

From our collective expertise and discussions at the recent National Mouse Genetics Network meeting, ‘CHD: From Cardiac Gene Variants to Mouse Models’, we recommend the following:

1. Improving the design of CHD genetic/genomic studies to include patients with accurate phenotyping, with a family history and, where possible, stratified subgroups of patients on the basis of their developmentally related CHD phenotypes.
2. Extending analysis to cover the non-coding genome by including splicing and/or structural variants in analysis of CHD patient data.
3. Developing a version of segmental analysis to describe CHDs in mice using the aligned terminology with that used to describe the human heart, including malformations linked to a simplified version of the CHD coding systems in International Classification of Diseases, Version 11 (ICD-11) and Human Phenotyping Ontology (HPO).
4. Expanding HPO cardiac and CHD terms to more closely align with ICD-11.
5. Accelerating the development of anatomical and gene atlases, allowing comparison of human and animal (mouse, zebrafish, etc.) cardiac morphology and gene expression patterns.
6. Extending Gene Ontology and/or other resources with up-to-date transcriptomic data from all cardiac cell types, across developmental windows and models.
7. Developing improved and more relevant assays for variant testing, both *in vitro* and in non-mammalian *in vivo* models, prior to the use of ‘gold standard’ mouse mutants.
8. Improving accessibility of resources for the design and creation of mouse mutants that model patient variants and, importantly, copy number variation, with follow-up access to specialist cardiac phenotyping platforms. This will help to accurately describe the anomaly and also help with standardisation of nomenclature.
9. Increasing efforts to highlight to clinicians – through journal editorials, articles and meetings – the importance of comprehensive description and coding of CHDs for research purposes.
10. Communicating advances in cardiac developmental biology to geneticists, cardiologists and patient groups in a format that is accessible, engaging and relevant to the issues they face with clinical diagnoses.

To facilitate 9 and 10, clinicians, geneticists and scientists should be brought together in joint meetings and endeavours. These could include, for example, the European Societies of Cardiology Working Groups on Development, Anatomy and Pathology; Adult Congenital Heart Disease; the Association for European Paediatric and Congenital Cardiology; and the Association for Inherited Cardiac Conditions.
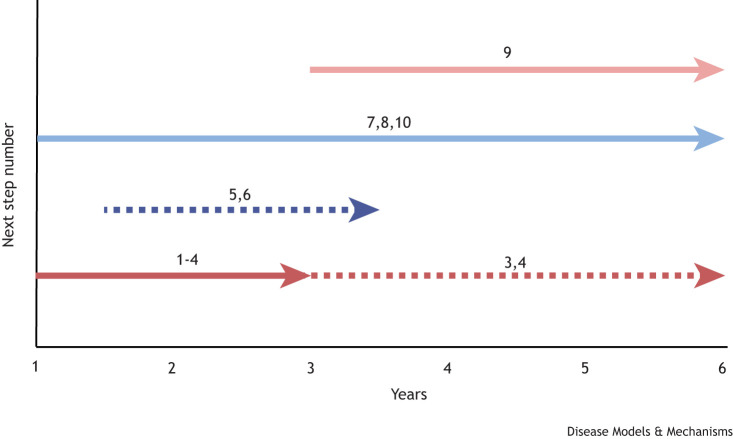

Estimated timeline for achievement of the next steps. Dotted lines indicate that the timing is less well defined than for solid lines.

## Filtering gene variants from CHD genetic studies

Appropriate filtering of the data from studies of CHD patients is a complex issue even when high quality genomic data are obtained. When carried out well, it whittles down a huge number of potential variants to a few biologically relevant ones that can be passed on for validation, thus providing a critical step in the pathway of variant analysis. At worst, it excludes relevant genes and/or inaccurately highlights genes for follow up.

**Fig. 1.**
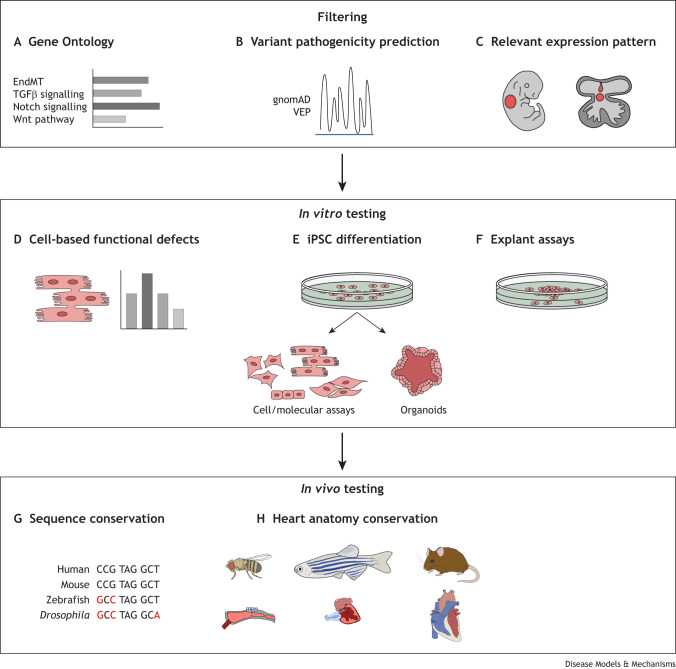
**Filtering and validating variants prior to testing in animal models.** (A-H) After standard bioinformatic filtering, many variants are likely to remain. In order to filter these to a number that is appropriate for functional testing, they should be passed through a second phase of filtering that includes Gene Ontology to determine whether they are associated with relevant biological and disease processes (A); analysis of whether the identified variants are predicted to be pathogenic using tools such as gnomAD and Variant Effect Predictor (VEP) (B); and gene expression analyses (either novel or using existing gene expression databases) that confirm that the gene is expressed in a pattern relevant to the specific congenital heart defect (CHD) (C). This should highlight a small number of potential variants that can proceed to *in vitro* validation testing using cell-based assays, in which the gene is disrupted and/or the variant is inserted to determine effects on cell behaviour (D); induced pluripotent stem cells (iPSCs), in which the effects of the gene variant can be seen on, e.g. differentiation capacity (E); and explant assays, in which the effect of disrupting gene function can be assessed in a CHD-relevant assay (F). Following confirmation of a potential role in cardiac cells *in vitro*, it is then appropriate to proceed to variant testing in an animal model. This model should be selected on the basis of gene sequence conservation (G) and on the similarity, between the animal model and human, in the structures of the heart that are relevant to the CHD (H). EndMT, endothelial-to-mesenchymal transition.

Population-scale genomic research into sporadic CHD has been made possible by GWAS, CNV analysis and, more recently, NGS. Although GWAS and CNV analyses have identified regions of the genome associated with CHDs, they rarely identify specific genes that can be pinpointed as the cause of disease in individual patients. By contrast, exome sequencing has been successful in identifying potential CHD-causing genes. These studies have shown that the majority of sporadic cases of CHD harbour numerous inherited or *de novo* single-nucleotide variants (SNVs) (Zaidi et al., 2013; Homsy et al., 2015; Sifrim et al., 2016; Page et al., 2019) that have to be filtered to identify one, or a small number, of the likely causative variants. For the first stage of filtering, which is applicable to all genomic studies irrespective of disease type, guidelines are available, that if followed, significantly improve the quality of the outputs (for examples, see Richards et al., 2015; ACGS Best Practice Guidelines for Variant Classification in Rare Disease, 2024; The Clinical Genome Resource, 2023) However, the second stage, which is disease specific, is much more variable, with a wide range of approaches and methodologies adopted. In filtering down to likely CHD-relevant candidates, there is a basic assumption that implicated genes play a role during cardiac development and thus must be expressed in the right tissues, at the right time, or else malformation occurs (Fig. 1). However, in the case of the heart, many genes play their essential roles in progenitor populations, such as the second heart field (SHF; Box 1) or neural crest cells (NCCs; Box 1), before they reach the heart. Thus, filtering gene variants on the basis of cardiac involvement is contingent on knowledge about the likely underlying developmental deficit that leads to the anomaly, and on the spatiotemporal expression pattern of the gene and the cell types involved. Some genomic studies have successfully used transcriptomic data from RNA sequencing (RNAseq; Box 1) of whole foetal mouse hearts as a filter dataset (e.g. Zaidi et al., 2013). For future studies, filter datasets (ideally human) should include transcriptomic datasets from early stages of heart formation, including progenitor populations, such as the SHF and NCCs, disruptions of which are a major cause of CHD, at least in mice (Stefanovic et al., 2021; Erhardt et al., 2021). An alternative to gene expression-based filtering is to use Gene Ontology (GO) analysis (Box 1) for informatic-based filtering. However, the manually curated gene lists used in this approach are limited and incomplete (Queen et al., 2023). For example, the recent release of 400 terms involving 138 genes related to valve development (Ahmed et al., 2023) is limited to well-known genes involved in valve development, rather than those that have been derived from valve-specific transcriptomic studies (e.g. MacGrogan et al., 2016; Hulin et al., 2019; Queen et al., 2023). Application of artificial intelligence (AI) to GO terms, with its potential to interrogate all available data resources in a very short period of time, is an obvious strategy to improve this and is likely to be useful in the future. Following these initial broad data filters, there will then need to be evaluation of expression patterns of the limited remaining genes, ideally within human tissues, as outlined above. Biobanks such as the Human Developmental Biology Resource (HDBR) and data resources such as the Human Developmental Cell Atlas make the analysis of specific gene expression patterns readily achievable.

Once gene filtering has been carried out, the pivotal question for genes confirmed as being developmentally relevant is whether variants are causal for specific malformations. In the first instance, it is important to determine whether variants in the candidate gene(s) have been previously associated with disease. Resources such as ClinVar and ClinGen are invaluable for this. ClinVar, for example, is a freely accessible, public archive of reports of human variations classified for diseases and drug responses, with supporting evidence. ClinVar thus facilitates access to and communication about the relationships asserted between human variation and observed conditions, and the history of those assertions. For assessing the likely pathogenicity of a variant, bioinformatic resources such as gnomAD and Variant Effect Predictor, although not perfect, are very useful for predicting the likelihood of pathogenicity of individual variants. Following such analyses, however, many will be classified as VUS and require further investigation (Fig. 1).

## Validating VUS: *in vitro* assays

As a first step to investigate variants in genes identified through genetic studies of CHDs, *in vitro* testing can provide experimental evidence that variants, including VUS, are pathogenic. Although many variants can be shown to lead to gene dysfunction in an *in vitro* assay, for example luciferase assays for transcription factors, this does not equate to causation of abnormal morphogenesis. Although cell lines for different cardiac cell types are available, these have the disadvantage of rarely matching the specific characteristics of the embryonic cell type of interest and cannot be used to study three-dimensional (3D) form as is relevant in the case of CHD. The use of *in vitro* models for modelling aspects of CHDs has been well reviewed recently (Hofbauer et al., 2021a,b; Rufaihah et al., 2021; Rao et al., 2022); here we discuss some of the key issues, expanding on some that we feel are particularly pertinent.

Human induced pluripotent stem cells (hiPSCs; Box 1) have been widely used to make cardiomyocytes, and it is also possible to generate hiPSC-derived endocardium, pacemaker-like cardiomyocytes, valve interstitial cells and smooth muscle cells, as well as the progenitors that give rise to them. However, these other cell types are less frequently employed, mainly because robust protocols have only recently become available (Neri et al., 2019; Liu et al., 2020a,b; Yechikov et al., 2020; Liu et al., 2023; Cai et al., 2024). hiPSC-based experiments also have the advantage that genetic background-matched controls can be engineered by CRISPR-Cas9 (Box 1) to correct the variant in patient-derived cells or in the reference KOLF2.1J cell lines (Pantazis et al., 2022). However, there are fundamental problems with the hiPSC approach for structural malformations. First, when differentiated cell types are generated from hiPSCs, the effects of different progenitors that may be responsible for the malformation are usually not explored. As an example, it is well established that disruption of the SHF, NCCs and the endocardium can result in common arterial trunk (Box 1; Bajolle et al., 2009). Thus, gene expression studies before differentiation of hiPSCs could guide the experimental approach. However, where the underlying cause of a malformation is unclear, it can be difficult to know what to differentiate hiPSCs to. As an example, hiPSCs have been used to model HLHS – a complex malformation characterised by a small left ventricle, stenosis or atresia of the mitral and aortic valves, and a hypoplastic ascending aorta. Of the 12 currently available papers that have used hiPSCs to study the aetiology of HLHS – for which there are at least three subtypes (Tchervenkov et al., 2006; Crucean et al., 2017; Moon et al., 2024) – ten papers have differentiated hiPSCs to cardiomyocytes, despite it being unknown whether a defect in the differentiation or function of the myocardium was the underlying problem in the patients from which the hiPSCs were derived. Interestingly, none of these studies have differentiated hiPSCs to valve cells, despite mitral atresia (Box 1) being speculated as being the initiating event for at least one of the subtypes of HLHS (Sedmera et al., 2005). Even if cardiomyocytes are produced and are the relevant cell type, there are significant uncertainties as to what type or maturity of cardiomyocyte has been produced.

Most importantly, heart morphogenesis is highly dependent on cell interactions, and the mechanical forces found in the developing heart are complex to mimic *in vitro*. The interpretation of outputs from cell-based assays, when the defects they are modelling are those of 3D architecture, poses obvious challenges. Endothelial-to-mesenchyme transition (EndMT) assays, in which the migration of endocardial cells into a collagen gel is assessed, have been widely used for modelling defects that affect the cardiac cushions (Box 1). In some instances, these may be highly relevant, for example if the gene under analysis is involved in EndMT (e.g. MacGrogan et al., 2016; Papoutsi et al., 2018). However, valve and septa formation is complex, with many different processes involved. Thus, assays for these other aspects are needed if genes involved in non-EndMT developmental processes are to be assessed. Cardiac organoids (also named cardioids) with multi-chamber structures are in development (Hofbauer et al., 2021a,b; Lewis-Israeli et al., 2021; Schmidt et al., 2023) and, if they can be developed to replicate important features of cardiac structure and function, may be useful for modelling CHDs in the future. Again though, considerable work needs to be done to improve the robustness and reproducibility of these models to reach this point of utility.

## Validating VUS: *in vivo* non-mammalian models

Having carried out appropriate *in silico* and *in vitro* studies, it then becomes important to test the relevance of the gene, or to model the variant, *in vivo* (Fig. 1). Modelling requires a human orthologue to be expressed and a CHD-relevant developmental process to be conserved. In addition, the gene sequence must also be conserved between human and the model organism. Thus, although non-mammalian models may be attractive for many reasons, and can be used to complement investigations in mice, lack of conservation of anatomy and sometimes genetics means that they are not always applicable. This said, a number of non-mammalian models have been used in recent studies, including the fruit fly, frog and chicken. For example, the fruit fly has been used for testing the developmental requirement of candidate CHD genes, for instance for genes linked to HLHS (Zhu et al., 2017; Ekure et al., 2021; Birker et al., 2023). However, the structural differences between non-mammalian and human hearts can make their utility questionable for studying complex morphogenesis. Although a detailed overview of this topic is beyond the scope of this Special Article, the topic has been reviewed recently (Rufaihah et al., 2021).

The fish heart has a separated atrium and ventricle, with valves separating these chambers and the outflow vessel, and resembles a simplified version of the mammalian heart. As a consequence, it is well recognised as a model system for studying cardiac development and has been used to model CHDs (recently reviewed in Grant et al., 2017; Yang et al., 2024). Most mammalian genes are represented in the zebrafish genome, although the teleost genome duplication event (Box 1; Taylor et al., 2001) may be an issue if there are duplicated and redundant genes. In most cases in which zebrafish have been used to model CHDs to date, either null mutants have been created (e.g. Huang et al., 2022) or, more commonly, antisense morpholino oligonucleotides (e.g. Ma et al., 2022; Liu et al., 2022) have been used to determine the relevance of the gene for heart development, although the latter approach is falling out of favour. Gene variants can rapidly be created in zebrafish using CRISPR-Cas9 technology, making this a particularly amenable model for these types of studies. However, it is usually considered important to compare the variant mutant with a complete null mutant for the gene, to evaluate the effect of the variant. In this context, it has been shown that genetic compensation can be activated in the presence of nonsense-mediated decay (Box 1), which alters the broad transcriptional landscape and thus the phenotype of the null mutant (reviewed in Sztal and Stainier, 2020). Genetic rescue experiments (Box 1) are of utility to test this, if appropriate controls are carried out. Although the zebrafish has proven utility for modelling highly conserved aspects of cardiogenesis, screening in the zebrafish is not likely to be of use for modelling more complex aspects of heart formation, for example validation of putative ASD or VSD genes, as there is no atrial or ventricular septum in fish. However, it may be appropriate for validating variants linked to arterial valve (Box 1) or outflow tract (Box 1) abnormalities that rely on the addition of SHF progenitors, as this process is conserved between fish and mammals (Zhou et al., 2011; Nevis et al., 2013). Thus, although zebrafish has been used successfully to test patient variants for some cardiac anomalies (e.g. Lessel et al., 2016; Farr et al., 2018; Derrick et al., 2024), for more complex malformations it will be necessary to use the mouse, with its close conservation of cardiac anatomy to human.

## Considerations when designing a mouse model for variant testing

For most purposes, the mouse is the organism of choice for modelling complex heart phenotypes. Given the evolutionary proximity between mouse and human, there is usually close similarity at the gene sequence and anatomical level between the two species. However, the faster development of the mouse embryo and the scarcity of human embryos available for gene expression studies can make it difficult to directly compare expression patterns between the two species. As mentioned earlier, the availability of human and mouse atlases with labelled heart sections (HDBR Atlas), and the development of detailed atlases of mouse and human *in situ* hybridisation (e.g. GenePaint), single-cell RNA sequencing (scRNAseq; Box 1) (Encyclopedia of DNA Elements) and proteome data (The Human Protein Atlas) will be valuable for carefully comparing mouse and human genes potentially involved in CHD.

Cardiovascular disease modelling in mice using gene targeting in embryonic stem cells is a time-consuming and resource-intensive process, with most lines generated as loss-of-function alleles for the gene of interest. This includes large international endeavours, such as the International Mouse Phenotyping Consortium (IMPC), that have created and phenotyped ∼9000 gene knockouts (International Mouse Knockout Consortium et al., 2007; Dickinson et al., 2016). In humans, many disease genes exhibit a dominant phenotype, causing disease in the heterozygous state (e.g. *TBX1*, *NOTCH1* and *GATA4*), such that a wild-type copy of the gene is also inherited. By contrast, this is not always the case in mice, where inactivating mutations in genes may replicate the patient phenotype only in the homozygous condition (e.g. *Tbx1*; Merscher et al., 2001; Jerome and Papaioannou, 2001). Importantly, the mouse null phenotype may be more severe, potentially leading to early embryonic lethality, so that a close comparison cannot be made between the mouse null and patient phenotype. It is also well established that the phenotype and penetrance of the CHD will vary with the genetic background of the mice into which the gene mutation is introduced. Partial penetrance of CHD phenotypes can vary significantly between human and mouse. For example, in a family with heterozygous mutations in *ROBO4* resulting in partially penetrant BAV and thoracic aortic aneurysm (Box 1) across three generations, 8/10 potential carriers of the mutation were symptomatic. By contrast, only 3% of heterozygote mice carrying the variant allele, and 11% of homozygotes, developed the expected phenotypes (Gould et al., 2019). Thus, penetrance was much reduced in the mouse model of the human variant compared to the human family. As a consequence, large numbers of animals (more than 25 homozygotes and 100 heterozygotes) may need to be screened to detect low-penetrance malformations. Although this variability is a disadvantage in some situations, and can lead to variation in findings between research studies, humans are not generally inbred, and the use of multiple genetic backgrounds of mice can lead to a better understanding of the spectrum of malformations that can result from the gene mutation, more closely mimicking what is seen in human CHD families. This can be of advantage for translational studies aimed at therapeutics (Olguín et al., 2022).

The standardised production and phenotyping protocols utilised by initiatives such as the IMPC have been invaluable for identifying new genes with roles in cardiac development and disease (Spielmann et al., 2022); however, few patient variants have been reproduced in mouse models. The advent of CRISPR-Cas9 genome-editing techniques allows streamlined generation of patient-modelled mutations. For example, for missense or small indel (Box 1) alleles, the design of repair templates harbouring the mutation of interest can allow these variants to be precisely introduced into the mouse genome, allowing the pathogenicity of the variant to be tested. Although the efficiency of genetic editing in mice is very high, evidence-led triaging of novel missense, frameshift and nonsense variants should be first evaluated in order to prioritise generation of mouse models. The technology allows testing of newly identified non-coding variants that potentially affect gene splicing (e.g. *MYBPC3* in hypertrophic cardiomyopathy), as well as more complex structural variants, which can be difficult to test *in vitro* as splicing can follow tissue-specific regulation.

After microinjection or electroporation of one-cell-stage embryos with editing reagents and subsequent transfer to foster mothers, offspring can be screened by PCR and sequencing. Founder mice containing the desired genetic change – for which they can be mosaic – are identified and bred. The genetic change, on target, is confirmed at the subsequent generation, and it is also important to check against additional/random integration of the donor template and potential off-target mutation linked to the locus of interest (Burgio and Teboul, 2020). Prior to phenotypic analysis, a few generations of backcrossing (after five backcross generations, <5% of the founder genome remain) with an inbred strain is recommended to breed out potential off-target mutagenesis events.

If the newly generated mouse line does not show a mutant phenotype in a heterozygous or homozygous setting, a ‘genetic sensitisation’ experiment can be carried out, as has been used extensively in *Drosophila*. This relies on breeding the new line (A) with a second one harbouring a heterozygous mutation in a gene (B) that is thought to be involved in the same process, hoping to favour the manifestation of a mutant phenotype in the double heterozygous mouse line (A/+;B/+). A pertinent example of this is the use of *Notch1* heterozygotes as a sensitising mutation for a range of cardiac phenotypes, including aortopathy (Box 1) and BAV (Koenig et al., 2015; Siguero-Álvarez et al., 2023; Tessler et al., 2023).

Larger structural variants such as aneuploidies, or insertions, deletions or complex rearrangements at sub-whole chromosome levels, are known to be a significant cause of CHDs, both syndromic and sporadic non-syndromic. Our ability to generate mice with duplications or absence of large chunks of chromosomes lags behind targeted deletion of genes or insertion of specific gene variants but continues to evolve. Success in the case of 22q11 deletion syndrome, where mice deleted for the key region of chromosome 22 helped to identify *Tbx1* as the key CHD-causing gene (Kimber et al., 1999; Taddei et al., 2001), and mouse models mimicking trisomy 21 that improved the understanding of CHD in Down syndrome (e.g. O'Doherty et al., 2005; Lana-Elola et al., 2024), are rare exceptions, as these studies have been labour intensive and time consuming. Advancements in mouse chromosome engineering are crucial if we are to understand how CNVs of entire or partial chromosome regions lead to CHDs. Evolving techniques, such as CRISpr MEdiated REarrangement (CRISMERE; Box 1; Schaeffer et al., 2023), raise the possibility that more structural variants can be modelled in the not-too-distant future.

## Phenotyping mouse models of CHD

**Fig. 2.**
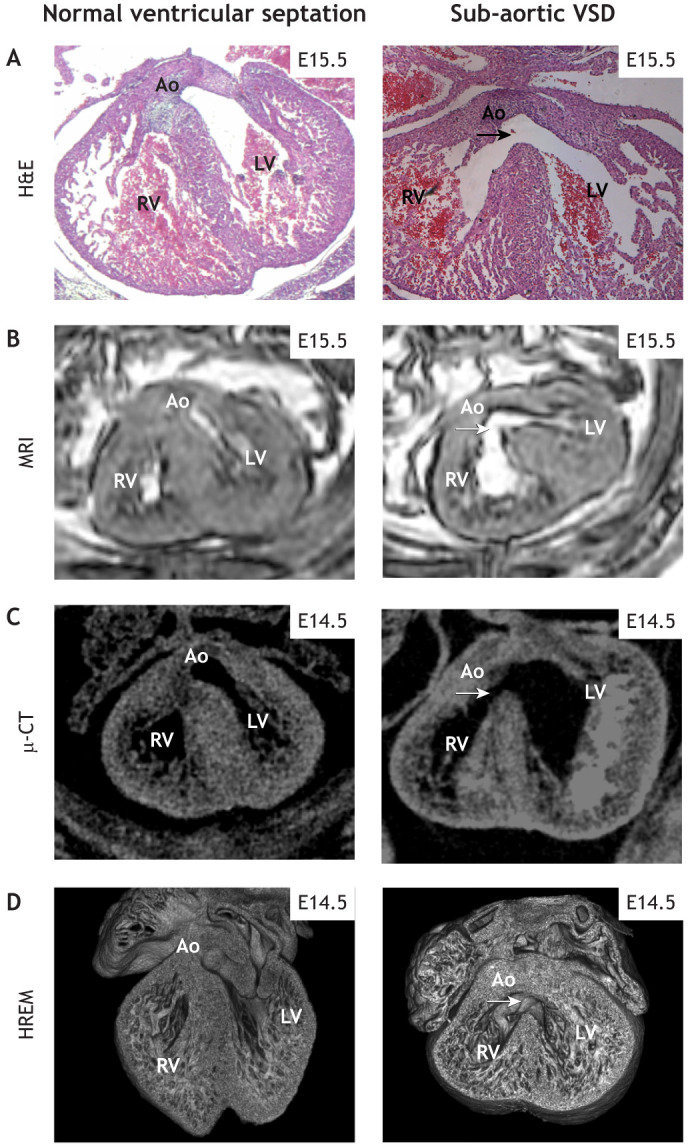
**Imaging normal and abnormal mouse heart development.** In each case, a normal heart (H&E and MRI images are at E15.5; µ-CT and HREM images are at E14.5) is shown on the left and a heart with a membranous VSD is seen on the right (arrows). (A) H&E. (B) MRI. (C) µ-CT. (D) HREM. Resolution is lower for the images obtained using µ-CT and MRI, although they, together with HREM, have the benefit that they generate 3D datasets. Ao, aorta; H&E, Haematoxylin and Eosin; HREM, high-resolution episcopic microscopy; LV, left ventricle; MRI, magnetic resonance imaging; RV, right ventricle; VSD, ventricular septal defect; µ-CT, micro-computed tomography.

It is essential that variant mice are phenotyped and described in a way that can be compared directly with human patients. At the same time, the very broad range of phenotyping methods that can be applied in model organisms can reveal subtle phenotypes that have not been, or perhaps cannot be, identified in humans. This, combined with the statistical power that comes from having multiple biological replicates with the same genotype, means that model organisms, and specifically mouse models, have an important role in establishing the morphogenetic mechanisms that underlie different types of CHD. These models can also be used to establish whether the CHD is associated with, or predisposes to, other malformations or adult diseases. The methods used to phenotype mutants will vary considerably, depending on whether they are carried out by individual research groups or collaborative initiatives such as the IMPC (Table 1). These studies are usually carried out at embryonic day (E)14.5-E15.5, when cardiac septation is complete and the adult structure of the heart is attained. In most individual research groups, sectioning and histological staining of mouse hearts, ideally in three orthogonal planes, is the standard way of phenotyping mouse mutants for CHDs (Fig. 2A). One major advantage of this technique is that embryos/foetuses can be examined at any stage of development, before it is possible to use other techniques (see below). In expert hands, and when complemented with 3D reconstruction of serial sections, this can produce high-quality data that can be compared to human imaging. However, as for all imaging techniques, it is limited by the skill and experience of the individual researchers to accurately interpret the data obtained. Despite this constraint, it will likely remain the cornerstone of CHD phenotyping in mice, and, thus, the development of sequential segmental analysis guidelines for the systematic analysis and description of the normal and abnormal heart in mice will be a major step forward.

**Table 1.**
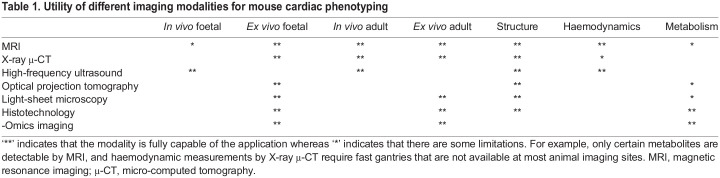
Utility of different imaging modalities for mouse cardiac phenotyping

The types of analysis possible within individual research groups contrasts with the large-scale phenotyping data produced by initiatives such as the IMPC, where a standardised global pipeline ensures that data are produced, recorded and delivered in the same way, independent of phenotyping centre. Imaging technology is an essential tool in phenotyping CHD mouse models, both for visual comparison to human images and for evaluating hemodynamic function and 3D morphometry that is central to the definitions of these conditions. High-frequency ultrasound is particularly powerful for phenotyping late gestation and adult mice, as the increased spatial resolution afforded by the higher sound frequency allows echocardiographic assessments that closely resemble those of their human counterparts. This includes measurements of heart morphometry, blood velocity time series and flow rates through major vessels, together with embryonic development monitoring throughout gestation. Incidentally, high-frequency ultrasound can be applied to models under anaesthesia or in an awake state, the latter potentially enabling direct translation to the patient setting (O'Riordan et al., 2023). These approaches have been successfully applied to describe novel phenotypes in adult (Assimopoulos et al., 2022) and foetal (Rahman et al., 2021) mice. Moreover, this technology is rapidly evolving, with techniques for visualising complex flow patterns that have been demonstrated in humans (Avesani et al., 2021) being translated for high-frequency/small-animal use. The establishment of guidelines for phenotypic assessment is indeed crucial for ensuring rigor and reproducibility, as well as increasing the potential for clinical translation.

Magnetic resonance imaging (MRI) is another powerful tool for cardiac phenotyping in live mice. Although the trade-off between resolution and scan time in this modality means that the image fidelity in mice does not at present match that obtained in humans (Fig. 2B), cardiac MRI has many of the same advantages in mice, including the ability to measure heart motion and blood flow in 3D (Russo et al., 2020). Moreover, the ability to assess heart wall structure is particularly relevant for discovering adult heart disease phenotypes (Cheng, 2022). MRI is also useful for the analysis of fixed tissue, allowing detailed and non-destructive assessment of heart morphology in foetal (from ∼E15.5) and adult mice. A related modality for the 3D morphological assessment of fixed specimens is X-ray micro-computed tomography (µ-CT), using which excellent soft-tissue contrast can be obtained with iodine staining (Handschuh and Glösmann, 2022), again from ∼E14.5-E15.5 (Fig. 2C). µ-CT has been used extensively by the IMPC to evaluate novel phenotypes in embryonic lethal mutations. Combined with whole-embryo computational analysis, this approach enables high-throughput screening for morphological phenotypes including cardiac defects. Earlier-stage embryos (<E11.5) can be assessed in a similar manner using 3D optical techniques, such as optical projection tomography (Sharpe, 2003) or light-sheet microscopy (Udan et al., 2014). High-resolution episcopic microscopy (HREM) is another alternative that provides even higher resolution (Fig. 2D), at the expense of longer imaging time (Weninger et al., 2018). HREM technology empowers researchers to leverage serial 2D aligned stacks of images, enabling the execution of 3D reconstructions. The 3D visualisation afforded by HREM facilitates a nuanced understanding of topology and morphology, surpassing the capabilities of traditional histological studies. Optical coherence tomography is another technique that can be used in early-stage embryos and enables *in vivo* assessment of flow and structure (Raghunathan et al., 2016).

An aspect of cardiac phenotyping in mice is that the number of animals requiring examination can be large, favouring high-throughput and automated methods. There is, at present, a deficit in computational methods that can identify cardiac phenotypes from 3D images in a manner similar to that taken by human anatomists. Whereas an anatomist may first describe the morphology, then the connections and, finally, the malformations in evaluating an abnormal heart, computational tools for identifying cardiac phenotypes from 3D images are generally formulated more abstractly. An example is the deformation-based morphometry approach (Gaser et al., 2001) that has been used to analyse embryos for the IMPC project (Wong et al., 2014). This ‘model-free’ approach compares images without knowing in advance what a heart should look like. However, these methods assume one-to-one anatomical correspondence between the embryos being compared, with no structures added or removed, and with the topology preserved. These assumptions are not met in complex examples of CHD; however, this method has proven effective in detecting gross abnormalities in foetal hearts. Overall, this approach is good at finding shape differences but misses or mis-interprets cases in which the connections are incorrect.

AI and machine learning are developing rapidly and bring together imaging diagnostics with automatic evaluations in patient data. Applications for models like mouse are not yet as sophisticated. In the computer vision literature, two broad strategies for image interpretation are feature-based methods (Martin-Isla et al., 2020) and model-based methods following quantification of cardiac indices (Zamzmi et al., 2022). Implementing the model-based solution to the problem of high-throughput screening of 3D heart images would involve creating a parameterised model of a normal heart and asking the algorithm to adapt this to match a given 3D image. Conversely, a feature-based approach would search the image for small regions that are highly recognisable parts of the anatomy. This feature-based approach is likely closer to the approach of a human anatomist, but one could imagine both approaches having value in detecting cardiac phenotypes. Once a working approach has been found, machine learning methods could aid in making both approaches more computationally efficient. A strength of combined 3D imaging and computational morphology approaches is their assessment of many different aspects of the cardiac phenotype from a single series of animals. This helps in reducing animal use, consistent with the Replace, Reduce and Refine (3Rs) principles for ethical use of animals in research. Advanced 3D imaging modalities, for example Lightweight Analysis of Morphological Abnormalities (LAMA), for use with µ-CT have been developed, although there is not currently the capacity to incorporate LAMA into the high-throughput embryo pipeline of the IMPC to automatically perform image pre-processing, registration, statistical analysis and segmentation of embryo images and successfully uncovered known and novel dysmorphology (Horner et al., 2021).

In the future, advancements in phenotyping platforms, coupled with refined computational methodologies, particularly leveraging machine learning techniques, are poised to revolutionise phenotypic data. These innovations hold the promise of significantly enhancing our capacity to establish a robust and comprehensive model that elucidates the intricate relationship between genes, genotypes and phenotypes in CHDs.

## Understanding the underlying mechanisms of CHDs

Once detailed phenotypes are determined, it becomes important to understand the mechanisms underlying the observed malformations, and how they arose over developmental time and space. For example, defects in a key cardiac progenitor population, the SHF, lead to a range of related phenotypes in mouse and zebrafish models. These include defects in the outflow tract (including the arterial valves), right ventricle, atrioventricular canal (Box 1) and atria (Mosimann et al., 2015; Nevis et al., 2013; Kelly et al., 2001. If a mouse model presents with any (or a combination of) these phenotypes, it would be useful to know whether and how the identified gene functions in the SHF, allowing linkage to other genes and signalling networks, and specific phenotypes associated with this lineage. This can be achieved by creating conditional knockouts of the gene in the SHF using appropriate Cre drivers – in this case *Mef2c-AHF-Cre* (Verzi et al., 2005) or *Isl1-Cre* (Yang et al., 2006). Nowadays, a wide variety of Cre drivers are available for all contributing lineages and major cell types in the developing mouse heart (Table 2), allowing cell type and temporal dissection of gene function across cardiac development. Together with analyses of cell behaviour, including proliferation, cell death, alterations in cell shape or fate, and changes in migration or patterning, these studies provide a good understanding of the roles a CHD gene may play during normal development.

**Table 2.**
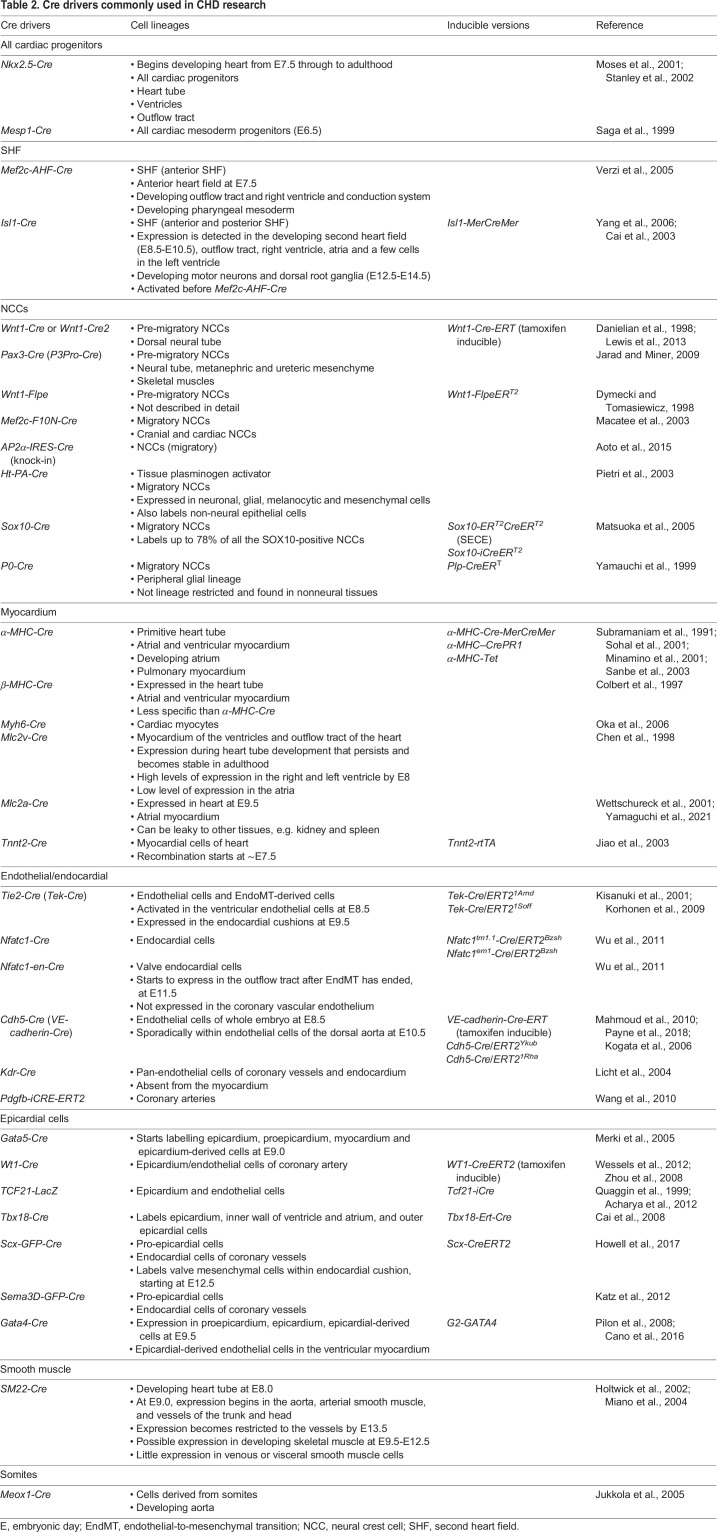
Cre drivers commonly used in CHD research

Recent progress in single-cell genomics and functional genomics, particularly through the use of cellular and animal models of CHDs, delivers fresh perspectives into the fundamental mechanisms of a CHD and its associated morbidities. Traditional bulk transcriptomic analyses provide in-depth mRNA coverage and an averaged representation of gene expression to be compared between wild-type and mutant samples. The limitation is that it disregards individual cell heterogeneity, cell identity and spatial distribution within a tissue. Although the transcript read depth is lower and less broad than in bulk transcriptomics, single-cell transcriptomics (scRNAseq) has emerged as a potent method for characterising cellular heterogeneity, rare cell types and disease state changes within a tissue. Recent advancements in analytical tools also enable the exploration of dynamic transition states during development, offering insights into lineage trajectories to some extent (Wagner and Klein, 2020). Additionally, this approach allows for speculation about cell-cell communications through ligand-receptor binding (Hou et al., 2020).

Although entirely novel insights have been rare when used in cardiac developmental biology, spatial transcriptomics (Box 1) technologies are rapidly advancing with varying degrees of resolution, as discussed by Asp et al. (2020). These technologies overcome the limitations of scRNAseq by facilitating large-scale, *in situ* profiling of RNA transcript distribution within tissue sections, revealing overall gene activity in a tissue sample and a map of where the activity occurs. The integration of individual cellular transcriptomes (Box 1) with their spatial context promises to revolutionise our understanding of biological processes and improve the phenotyping of animal models of CHDs. Spatial transcriptomics technologies have been successfully applied to human foetal stages (Cui et al., 2019; Asp et al., 2019; Queen et al., 2023), playing a potentially crucial role in addressing questions related to the spatial molecular heterogeneity of cardiac cells in normal and diseased hearts. Powerful advances in spatial proteomics to preserve important spatial information are emerging with antibody panel-based imaging mass spectrometry used for profiling human CHD samples (Hill et al., 2022). Alternately, Deep Visual Proteomics allows imaging and laser capture of features of interest on a tissue section for single-cell proteomics to explore cell heterogeneity within tissue architecture (Mund et al., 2022). This can be combined with advanced tissue-clearing techniques and machine learning to analyse thick samples, capturing small regions of interest, of ∼60 cells. Indeed, the technology has been applied on a whole human heart to explore tissue heterogeneity in early aortic plaques (Bhatia et al., 2022).

In summary, the technology that is supporting the phenotyping of mouse models of CHDs are advancing at a rapid rate, and this is likely to continue in the coming years. By closing the circle between what can be done in human patients and in mouse models of patient phenotypes, there are huge opportunities for better understanding the genetic mechanisms that underpin CHDs and feeding that back into patient care.

## Conclusions

**Fig. 3.**
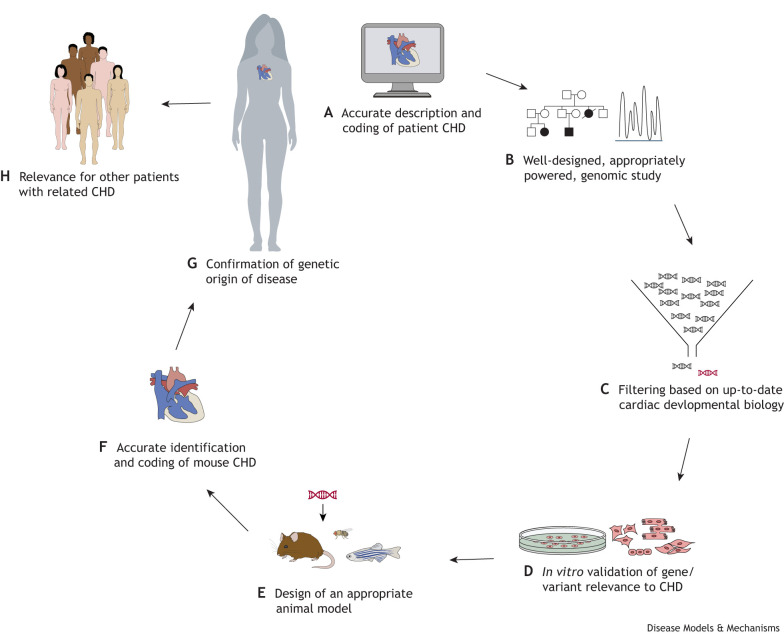
**Illustration summarising the ideal progress of variant identification and validation for CHD patients.** (A) A CHD is accurately described and coded in the patient's medical records, together with other relevant medical/family history. (B) Well-designed and appropriately powered genomic studies are carried out, ideally focused on patients with a related CHD. (C) Filtering based on up-to-date information from developmental biology and stratifying variants based on relevance to heart development. (D) *In vitro* gene/variant testing is carried out to gain evidence of variant pathogenicity or a functional role for the gene/variant in cardiac cells. (E) *In vivo* variant testing in an animal model appropriate to the type of CHD observed in the patient. (F) Accurate cardiac phenotyping of the mouse model. The description needs to be directly relatable to the patient CHD. (G) Genetic diagnosis for the patient that allows disease stratification and genetic counselling. (H) Information available to be used for better genetic diagnosis of CHD in the wider population.

Advances in cardiac developmental biology have the potential to improve genetic diagnosis of CHDs and improve patient care (Fig. 3). Better communication between cardiac developmental biologists, geneticists and clinicians will be essential if these advances are to be integrated into planning, conducting and interpreting genetic studies with CHD patients. Improvements in the design and production of mouse models that accurately recapitulate human disease, coupled with ‘humanised’ phenotyping, will allow validation of data from genetic studies and aid the understanding, and potentially treatment, of human CHDs.

Following our collective expertise and discussions at the recent National Mouse Genetics Network meeting, ‘CHD: From Cardiac Gene Variants to Mouse Models’, we have developed a series of recommendations, some of which, for example the mouse version of segmental analysis, are already in progress (Box 2).
